# Sensitive mass spectrometric determination of kinin-kallikrein system peptides in light of COVID-19

**DOI:** 10.1038/s41598-021-82191-7

**Published:** 2021-02-04

**Authors:** Tanja Gangnus, Bjoern B. Burckhardt

**Affiliations:** Institute of Clinical Pharmacy and Pharmacotherapy, Heinrich Heine University, Universitaetsstr. 1, 40225 Düsseldorf, Germany

**Keywords:** Biomarkers, Respiratory tract diseases

## Abstract

The outbreak of COVID-19 has raised interest in the kinin–kallikrein system. Viral blockade of the angiotensin-converting enzyme 2 impedes degradation of the active kinin des-Arg(9)-bradykinin, which thus increasingly activates bradykinin receptors known to promote inflammation, cough, and edema—symptoms that are commonly observed in COVID-19. However, lean and reliable investigation of the postulated alterations is currently hindered by non-specific peptide adsorption, lacking sensitivity, and cross-reactivity of applicable assays. Here, an LC–MS/MS method was established to determine the following kinins in respiratory lavage fluids: kallidin, bradykinin, des-Arg(10)-kallidin, des-Arg(9)-bradykinin, bradykinin 1-7, bradykinin 2-9 and bradykinin 1-5. This method was fully validated according to regulatory bioanalytical guidelines of the European Medicine Agency and the US Food and Drug Administration and has a broad calibration curve range (up to a factor of 10^3^), encompassing low quantification limits of 4.4–22.8 pg/mL (depending on the individual kinin). The application of the developed LC–MS/MS method to nasal lavage fluid allowed for the rapid (~ 2 h), comprehensive and low-volume (100 µL) determination of kinins. Hence, this novel assay may support current efforts to investigate the pathophysiology of COVID-19, but can also be extended to other diseases.

## Introduction

In March 2020, the World Health Organization declared a pandemic of coronavirus disease 2019 (COVID-19) due to alarming levels of spread and severity. COVID-19 is caused by infection with the novel severe acute respiratory syndrome coronavirus 2 (SARS-CoV-2). Hospitalized patients commonly present with symptoms of fever, cough, and dyspnea and can develop pulmonary edema in early disease^1^. It has been postulated that these symptoms are in connection with a dysregulated kinin-kallikrein-system (KKS)^2–5^. As yet, concentrations of peptides within the KKS have not been reported in COVID-19 patients and their role remains unclear.

**Figure 1:**
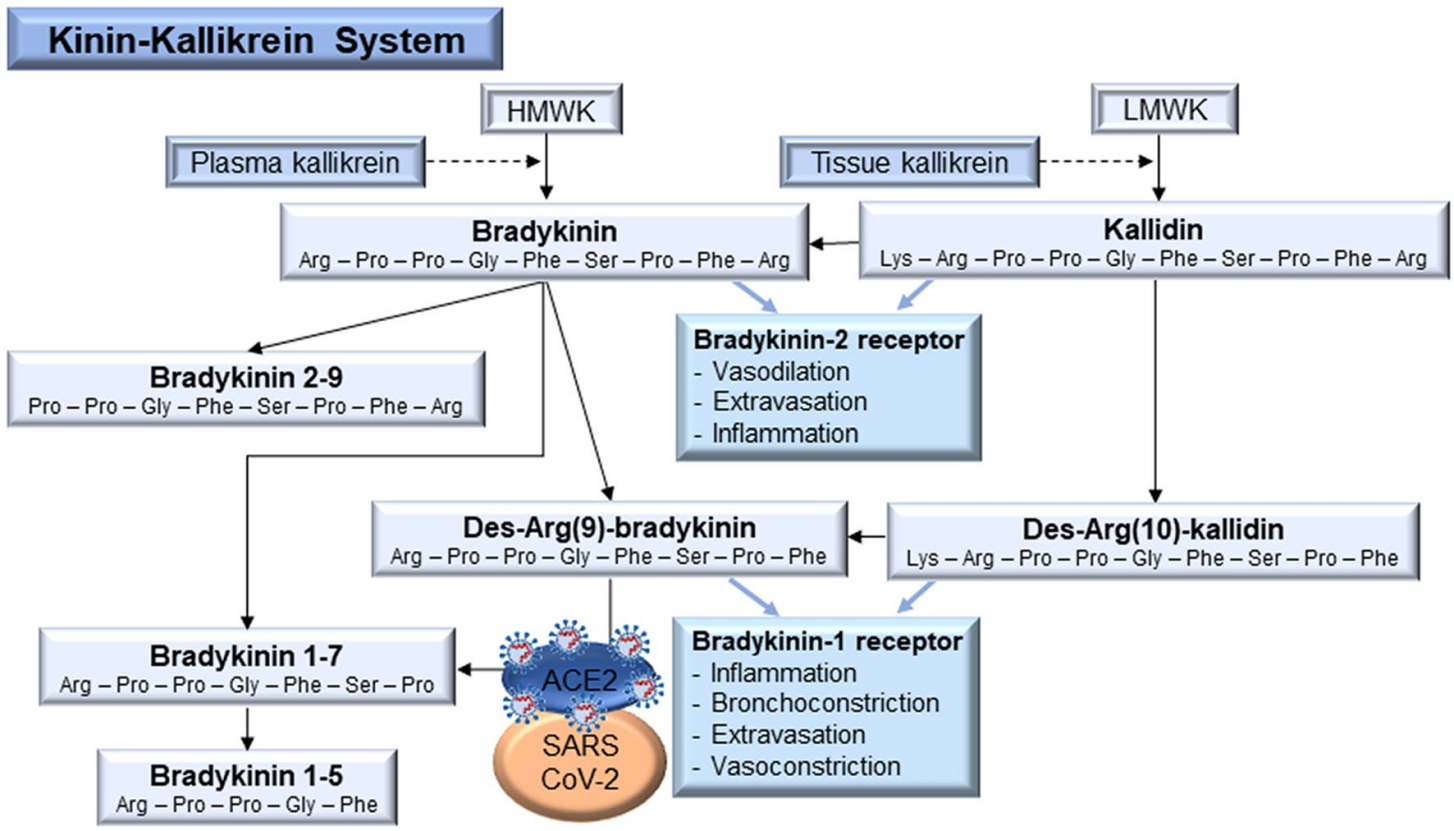
The kinin-kallikrein system and postulated connection with coronavirus disease 2019. ACE 2: angiotensin-converting enzyme 2, HMWK: high molecular weight kininogen, LMWK: low molecular weight kininogen, SARS-CoV-2: severe acute respiratory syndrome coronavirus 2.

Activation of the KKS, i.e. by tissue and plasma kallikrein, leads to the formation of the kinins bradykinin and kallidin (lys-bradykinin), both potent activators of bradykinin-2 receptors on endothelial cells^6^. The activation of these receptors promotes vasodilation, inflammation, and capillary leakage leading to edema (Fig. 1)^6,7^. Bradykinin and kallidin are cleaved by carboxypeptidase N and M into des-Arg(9)-bradykinin and des-Arg(10)-kallidin, respectively, which are ligands for the bradykinin-1 receptor, as demonstrated in in vitro experiments using human tissues^6,8^. The in vivo activation of bradykinin-1 receptors in animals was shown to mediate inflammation, bronchoconstriction, and extravasation, which causes (pulmonary) edema^9,10^. The receptor is furthermore upregulated during inflammation, thus providing increased receptor binding sites for des-Arg(9)-bradykinin and des-Arg(10)-kallidin^6,7,11^. While des-Arg(10)-kallidin is further cleaved into des-Arg(9)-bradykinin, the latter is mainly degraded by the angiotensin-converting enzyme (ACE) 2^12,13^. ACE 2 has been identified as the binding site of SARS-CoV-2, enabling it to enter cells^4,14^. It is thus assumed that cleavage of active des-Arg(9)-bradykinin into the inactive bradykinin 1-7 is considerably reduced during SARS-CoV-2 infection. In this context, viral attenuation of ACE 2 activity contributed to the pathogenesis of lung inflammation that was concomitant with increased bradykinin-1 receptor expression^12^. Therefore, the KKS is suggested to be involved in the pathogenesis of COVID-19 via the viral blockade of ACE 2, leading to elevated active des-Arg(9)-bradykinin levels (Fig. 1)^4,15^. Monitoring of seven kinin peptides (bradykinin, kallidin, des-Arg(9)-bradykinin, des-Arg(10)-kallidin, bradykinin 1-7, bradykinin 1-5 and bradykinin 2-9) may provide insights into the hypothetically altered kinin metabolism during SARS-CoV-2 infection.

Because ACE 2 is highly expressed in the nasal epithelium, the nose is presumed to represent the main entry point of SARS-CoV-2 prior to further spread within the host^16^. Therefore, saline lavage fluids from the respiratory tract would likely be the most suitable matrix to investigate alterations in the KKS peptide levels, as these fluids originate from the main area of viral infection and clinical symptoms in mild to severe courses (e.g. cough, nasal congestion, pulmonary inflammation and edema)^5,17^. While sampling of epithelial lining fluid in the lower airways by bronchoalveolar lavage is limited by its invasiveness requiring anesthesia, the use of nasal lavage fluid (NLF) offers the advantage of non-invasive, cheap and easy sampling^18^. In the NLF of healthy volunteers, kinin levels were reported to be typically less than 100 pg/mL by immunometric detection, however, no quantitative differentiation of the kinins was possible due to the underlying analytical technique applied^19,20^. The susceptibility to cross-reactivity with similar structures—which is the case for kinin peptides (Fig. 1)—represents the main disadvantage of immunoassays. Nevertheless, highly sensitive immunoassay-based quantification methods that can distinguish kinin peptides have been developed using plasma or tissues, but they require extensive effort for sample purification, including (multiple) solid-phase extractions (SPEs), liquid–liquid extraction, and chromatographic separation prior to immunoassay^21,22^.

Liquid chromatography coupled to tandem mass spectrometry (LC–MS/MS) represents a rational choice to overcome immunoassay-related limitations. Nevertheless, to date, no LC–MS/MS method has been reported that can comprehensively determine kinin peptides in respiratory saline lavage fluids. Validated LC–MS/MS methods are available for the determination of single kinin peptides (bradykinin, des-Arg-(9)-bradykinin and bradykinin 1-5) from plasma, serum, or whole blood and lack sensitivity in the desired low pg/mL range^23–25^. Owing to the substantial dilution of epithelial lining fluid by a factor of 60–120 during lavage, a suitable assay must be highly sensitive^26^. The reliable analysis of low levels of endogenous peptides in diluted matrix, whereby the peptides are often characterized by non-specific peptide adsorption, requires extensive method development^27,28^. For that, design of experiments (DoE) has proven its usefulness as a lean tool for method development of multifactor-dependent settings and contributes to signal increase in LC–MS/MS^29,30^.

Therefore, this study aimed to develop and validate a novel LC–MS/MS method characterized by a broad calibration curve range to comprehensively and sensitively determine KKS peptides (bradykinin, kallidin, des-Arg(9)-bradykinin, des-Arg(10)-kallidin, bradykinin 1-7, bradykinin 1-5 and bradykinin 2-9) to enable reliable insights into their alterations in COVID-19—or other disease states (e.g. allergies or lung cancer)—in comparison to controls. Information regarding these alterations will contribute to the understanding of the pathophysiology related to these peptides and may help to identify new therapeutic targets.

## Materials and methods

### Chemicals and reagents

Kallidin trifluoroacetic acid (TFA) salt (96.9%, HPLC; Tocris, Bristol, UK), bradykinin acetate (99.0%, HPLC; Sigma-Aldrich, St. Louis, MO, USA), and their metabolites des-Arg(9)-bradykinin acetate (98.7%, HPLC; Santa Cruz Biotechnology, Dallas, TX, USA), bradykinin 1-7 TFA salt (≥ 95.0%, HPLC; GenScript, Piscataway Township, NJ, USA), bradykinin 1-5 TFA salt (≥ 95.0%, HPLC; GenScript), bradykinin 2-9 TFA salt (≥ 95.0%, HPLC; GenScript), and des-Arg(10)-kallidin TFA salt (95.9%, HPLC) were used in this study. [Phe^8^Ψ(CH-NH)-Arg^9^]-bradykinin TFA salt (97.5%, HPLC) was applied as the internal standard. Formic acid (FA, ≥ 98%) and TFA (100.3%) were supplied by Sigma Aldrich. HPLC-grade methanol, water, and dimethyl sulfoxide (DMSO, ≥ 99.9%), and MS-grade methanol and ammonium acetate (99.5%) were obtained from Fisher Scientific (Loughborough, UK). Furthermore, HPLC-grade acetonitrile (ACN, Applichem, Darmstadt, Germany), MS-grade water (Honeywell Fluka, Seelze, Germany), and ammonia (30.9%) (VWR Chemicals, Radnor, PA, USA) were utilized. Isotonic saline solution 0.9% was provided by B. Braun (Melsungen, Germany).

Sampling of NLF in healthy volunteers was performed in compliance with the ethical principles of the Declaration of Helsinki and was approved by the ethics committee of the medical faculty at the Heinrich Heine University of Duesseldorf in October 2017 (study number: 6112). Written informed consent was obtained from all participants before enrolment. Bioanalysis was conducted in accordance with Good Clinical Laboratory Practice.

### Preparation of stock and working solutions

Lyophilized kinin peptides were dissolved and diluted separately in 0.3% TFA in 25/75 ACN/water (v/v/v) prior to the preparation of a combined working solution containing 500 ng/mL of each peptide salt. [Phe^8^Ψ(CH-NH)-Arg^9^]-bradykinin as an internal standard was dissolved in 0.1% FA in water (v/v) and subsequently diluted to achieve a working solution of 500 ng/mL in 0.3% TFA in 25/75 ACN/water (v/v/v). All peptide solutions were prepared using low protein-binding tubes (Sarstedt, Nümbrecht, Germany).

### Sample preparation

A 0.9% isotonic saline solution was used as blank surrogate matrix for the respiratory saline lavage fluids. Owing to the endogenous presence of kinins and the long half-life of bradykinin 1-5, no reliable kinin-free human blank matrix could be generated. Optimized inhibitors were applied to effectively prevent the generation and degradation of the kinin peptides, based on previously published suitable inhibitor cocktails^31^. SPE was performed by applying 96-well Oasis® weak cation exchange (WCX) µ-elution plates (Waters, Milford, MA, USA). All cartridges were conditioned with 200 µL of methanol, followed by 200 µL of 5% aqueous ammonium hydroxide (v/v). Subsequently, the wells were prefilled with 150 µL of 3 ng/mL internal standard in 5% aqueous ammonium hydroxide (v/v), and 100 µL of sample was then loaded. Washing was performed with 300 µL of 5% aqueous ammonium hydroxide (v/v) and 300 µL of 10% methanol in water (v/v). Elution was conducted three times with 50 µL of 1% TFA in 75/25 ACN/water (v/v/v). The resulting eluates were evaporated to dryness under a gentle stream of nitrogen at 60 °C while shaking at 300 rpm. The residues were dissolved in 75 µL of 10/10/80 FA/methanol/water (v/v/v).

### LC–MS/MS

Chromatography was performed on an Agilent 1200 SL series system (Agilent Technologies, Ratingen, Germany) consisting of a degasser (G1379B), a binary pump SL (G1379B) and a column oven TCC SL (G1316B). A Phenomenex Synergi™ 2.5 µm Hydro-RP 100 Å column (100 × 2.0 mm; Torrance, CA, USA) was used for the chromatographic separation. The mobile phases were composed of water and methanol (B) both containing 3.2% DMSO and 0.1% FA (v/v). A 7.5 min binary gradient was applied, maintaining the amount of mobile phase B at 5% for 1.5 min, increasing it to 20% until 2.2 min, to 27% until 2.7 min, to 35% until 3.1 min, and finally to 95% after 6.2 min. Mobile phase B was kept constant at 95% until 6.7 min before decreasing it to 5% and column re-equilibration for 3 min. The flow rate was set to 400 µL/min, and the injection volume of 50 µL was applied with an HTC PAL autosampler (CTC Analytics AG, Zwingen, Switzerland). After every injection, the autosampler syringe was rinsed twice with 0.2% FA in 80/20 ACN/water (v/v/v). Samples were stored at 18 °C in the autosampler.

The LC system was coupled to an API 4000 (AB Sciex, Darmstadt, Germany) mass spectrometer equipped with a Turbo V source for detection. The electrospray interface was operated in positive mode with multiple reaction monitoring mode. The curtain gas was maintained at 31 psi, the collision gas at 8 psi, the nebulizer gas at 45 psi, the heater gas at 65 psi, the ion spray voltage at 5500 V, and the source temperature at 350 °C. Peptide-specific parameters are displayed in Table 1.

**Table 1:** Peptide specific transitions and voltage parameters for mass spectrometric detection.

Analyte	Transition [m/z]	Dwell time [ms]	Declustering potential [V]	Entrance potential [V]	Collision energy [V]	Collision cell exit potential [V]
Kallidin	396.9→506.3	65	95	9	23	14
Bradykinin	530.9→522.4	65	120	10	31	14
Des-Arg(9)-bradykinin	452.8→263.2	75	85	10	22	15
Des-Arg(10)-kallidin	516.8→752.5	65	100	10	29	11
Bradykinin 2-9	452.8→404.3	50	120	9	24	11
Bradykinin 1-7	379.3→642.4	75	63	10	16	10
Bradykinin 1-5	287.2→408.3	50	61	8	15	11
[Phe^8^Ψ(CH-NH)-Arg^9^]-bradykinin	523.9→274.3	75	100	12	48	18

ms: milliseconds, m/z: mass-to-charge ratio, V: volt.

Data acquisition was conducted using Analyst^®^ 1.6.2 software (AB Sciex, Darmstadt, Germany), and raw data evaluation was performed using Multiquant™ 3.0.2 (AB Sciex, Darmstadt, Germany).

### Method development

#### Adaption of optimized injection solvent and sample collection material

A previously conducted DoE approach to optimize the injection solvent conjointly with the sample collection material to reduce non-specific adsorption of bradykinin and thus increase sensitivity^30^, had to be adapted to avoid peak broadening or breakthrough of the more hydrophilic kinin peptides. By using the D-optimal optimization model, an amount of 5–20% organic fraction in the injection solvent was investigated. This range correlated with the binary gradient, as no breakthrough of the kinins was expected. Furthermore, the calculations included a maximum intensity loss of 15% of the predicted intensity of the optimized injection solvent, based on current bioanalytical guidelines for the accuracy limits^32^. Six injection solvent compositions were calculated with distinct organic fractions and analyzed in triplicates by LC–MS/MS measurement, and responses and peak shapes were compared to the original injection solvent for bradykinin only (8.7% FA in 5.3/36.6/49.4 methanol/DMSO/water (v/v/v/v)).

#### Improvement of SPE recovery

The method development focused on maximizing recovery to reduce peptide loss during washing steps and to enable the detection of endogenous concentrations in the low pg/mL range. A previously developed SPE protocol for bradykinin only^30^ had to be adapted, since the kinin peptides differ in their amounts of hydrophobic and positively charged amino acids (Fig. 1). Mixed-mode strong cation exchange (MCX) and WCX µelution SPE were considered to evaluate which material would fit best to all analytes. All experiments investigating the washing and elution solvents were conducted using neat solution in duplicate.

### Validation

Bioanalytical method validation was conducted considering the regulatory bioanalytical guidelines of the US Food and Drug administration^32^. Linearity, accuracy, precision, sensitivity, recovery, matrix effect, carry-over, and stability were addressed during the validation process.

#### Linearity

Linearity was determined in six runs using nine to eleven distinct calibrator levels (depending on the lower limit of quantification (LLOQ) of the individual peptide), which were analyzed in single determinations. In compliance with bio-analytical guidelines, the actual concentration of 75% of all calibration curve standards had to deviate less than ± 15% (± 20% at the LLOQ) from their nominal concentration (relative error (RE))^32^.

#### Accuracy, precision and sensitivity

Accuracy and precision were assessed using up to seven quality control (QC) levels covering the whole calibration range on three distinct days. The number of QCs depended on the magnitude of the calibration range per peptide. Five replicates per QC level were analyzed each day. Accuracies were determined as the deviation of the actual concentration from the nominal concentration (RE) for within-run and for between-run accuracy. Using one-way ANOVA, within-run precision was calculated as repeatability and between-run precision as day-different intermediate precision (coefficient of variation (CV)). In line with regulatory guidelines, accuracy and precision were not allowed to exceed ± 15% (± 20% at the LLOQ)^32^. The signal-to-noise ratio (S/N) had to be higher than 5:1 at the LLOQ. The limit of detection (LoD) was further calculated as follows using the results from six calibration curves Eq. (1)^33^:

1 $$Limit \;\;of \;\;detection=\frac{3.3\sigma }{S}$$

Calculation of limit of detection. σ: standard deviation of the y-intercept, S: mean slope of the calibration curves.

#### Carry-over

Carry-over was evaluated by alternatingly injecting blank samples and upper limit of quantification (ULOQ) standards six times. According to regulatory bioanalytical guidelines, carry-over in the blank sample following the ULOQ was not allowed to exceed 20% of the LLOQ signal and 5% of the internal standard signal^32^.

#### Recovery and absolute matrix effect

Recovery was determined at four distinct QC levels covering the calibration curve range (high, middle, low and around the LLOQ) in triplicate. Pre-spiked extracted samples were compared to processed blank samples spiked with the same concentrations after µelution SPE. Matrix effects of the peptides were analyzed at the same four distinct QC levels by comparison of post-spiked samples to neat solutions (n = 3).

#### Stability

Stability studies were conducted at four QC levels (high, middle, low and around the LLOQ) under different storage conditions. Benchtop stability was investigated by placing the prepared QC samples at room temperature for one and three hours (n = 5). Freeze–thaw stability (room temperature to − 80 °C) was evaluated by analyzing the QC levels after one and three cycles (n = 5). Between each cycle, samples were frozen for at least 12 h. The autosampler stability was assessed by keeping QC samples in the autosampler at 18 °C for 18 h and then repeating the QC sample measurement. Finally, short-term stability of processed and evaporated samples was analyzed after storage for 24 h at + 4 °C (n = 5). Stability at the specific conditions was proven if the mean concentration at each level did not exceed ± 15%. To evaluate the stability of the analyte working solution, peak areas of the kinin peptides on 15 distinct days after 15 freeze–thaw cycles, measured routinely during method performance qualification (1 ng/mL, neat solution), were analyzed for their CV (acceptance criterion ≤ 15%).

### Applicability

Nasal lavage with 10 mL of 0.9% isotonic saline (5 mL per nostril) was performed in nine healthy volunteers (6 female/3 male). Volunteers were healthy adults above the age of 18 years without any signs of a respiratory infection or allergy. The volunteers were asked to tip their head backwards, hold the breath, and refrain from swallowing. The obtained fluid was collected directly into the inhibitor cocktail and was immediately vortexed after completing the sampling. The recovered volume was documented to enable the non-invasive normalization of kinin levels. At least 30% of the instilled volume had to be recovered during the lavage in line with the American Thoracic Society guideline for bronchoalveolar lavage^34^. The samples were centrifuged at 4 °C for 15 min at 500×*g* to remove cells, mucus, and debris. A 100 µL aliquot of the supernatant was analyzed.

## Results

### Method development

#### Improvement of sensitivity

**Figure 2:**
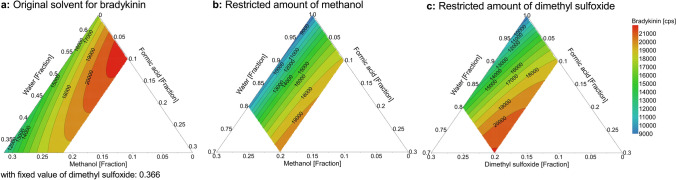
Contour plots of the D-optimal optimization for the response of bradykinin in distinct injection solvent compositions. In comparison to the contour plot including the original injection solvent for bradykinin with a fixed fraction of 0.366 dimethyl sulfoxide (**a**), restriction of the organic amount in context with the comprehensive determination of seven kinin peptides (**b** and **c**) lead to a shift to highest compatible organic and acidic fractions with regard to the hydrophilic peptides. White areas indicate regions, that were not investigated or were excluded due to peak distortion for hydrophilic peptides. cps: counts per second.

Analyzing the contour plots of the D-optimal optimization model for the original injection solvent composition and the contour plots with restricted organic amount, showed clearly that high acidic amounts were necessary for the injection solvent, as well as the highest organic amount that would be compatible with peak shapes of the hydrophilic peptides (Fig. 2). Of the six investigated injection solvent compositions, 10/10/80 FA/methanol/water (v/v/v) gave best peak shapes for bradykinin 1-5, by maintaining good signal intensity for bradykinin with 98.8 ± 2.3% of the optimized injection solvent composition (predicted: 91.4% with a log(D) of − 0.49 and probability of failure of 0.12%).

#### Improvement of recovery

**Figure 3:**
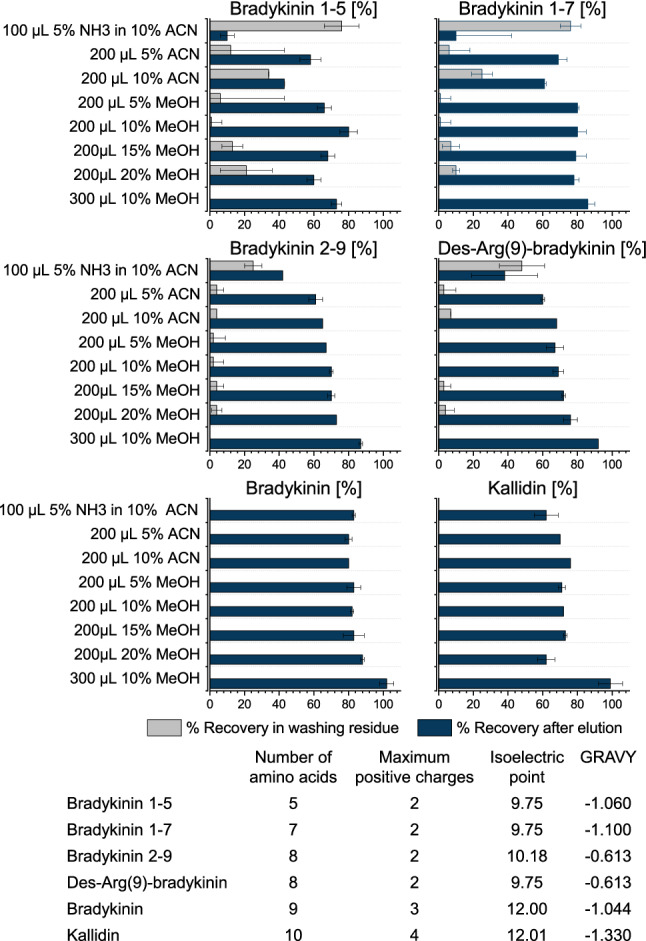
Recoveries during evaluation of washing steps using neat solutions and applying weak cation exchange µelution solid-phase extraction (n = 2). Peptide characteristics were calculated using ProtParam^43^. ACN: acetonitrile, GRAVY: grand average of hydropathicity index, MeOH: methanol, NH_3_: ammonium.

Using MCX SPE, the more hydrophobic or charged peptides (bradykinin, des-Arg(10)-kallidin, kallidin, and the internal standard) could not be satisfactorily eluted, when applying two 100 µL elutions of 5% of ammonium hydroxide in ACN, as indicated by low recoveries of 0–3%. Increasing the elution volume or adding ammonium acetate to the elution solvent to produce a salting-out effect did not substantially improve recoveries. Therefore, a customized protocol using WCX with modified washing steps was developed. In particular, the more hydrophilic kinins and those containing fewer amino functional groups (bradykinin 1-5 and bradykinin 1-7) were not robust regarding the recovery, as they were washed out when using an organic amount exceeding 10% methanol, subsequently affecting sensitivity and precision (Fig. 3). An increased amount of washing solvent (300 µL of 10% methanol) did not affect peptide recoveries but resulted in more precise values and was subsequently applied as described in the sample preparation section.

### Method validation

The results for linearity measurements gave best fits using quadratic regression except for bradykinin 1-5, where linear regression was applied (mean r ≥ 0.9960 for all analytes). The broad dynamic calibration curve ranges were (depending on the analyte) between 4.4 and 22.8 pg/mL for the LLOQ and between 4505.9 and 8255.5 pg/mL for the ULOQ. More detailed results for the linearity and the respective calibration curve ranges are presented in Table 2 and an example in supplementary Figure S1.

**Table 2:** Results for the assessment of linearity (n = 6) with corresponding peptide-specific nominal concentrations of the LLOQ and ULOQ.

Analyte	Linearity			Dynamic range	
	Mean r	Regression	Weighting	LLOQ [pg/mL]	ULOQ [pg/mL]
Kallidin	0.9998	Quadratic	1/x	4.4	4505.9
Bradykinin	0.9987	Quadratic	1/x^2^	6.7	6861.3
Des-Arg(9)-bradykinin	0.9981	Quadratic	1/x^2^	7.3	7419.1
Des-Arg(10)-kallidin	0.9987	Quadratic	1/x^2^	10.6	5348.9
Bradykinin 2-9	0.9977	Quadratic	1/x^2^	8.1	8255.5
Bradykinin 1-7	0.9980	Quadratic	1/x^2^	6.5	6669.0
Bradykinin 1-5	0.9960	Linear	1/x^2^	22.8	5757.0

LLOQ: lower limit of quantification, ULOQ: upper limit of quantification.

**Figure 4:**
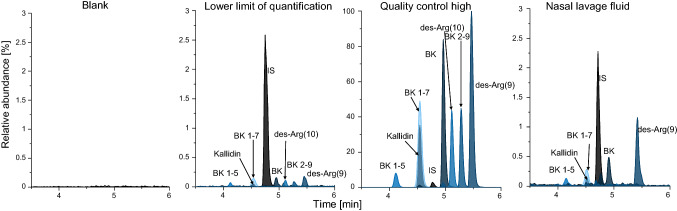
Representative chromatograms for the kinin peptides. The nasal lavage fluid was obtained in a healthy volunteer. BK: bradykinin, des-Arg(9): des-Arg(9)-bradykinin, des-Arg(10): des-Arg(10)-kallidin, IS: internal standard.

The within-run and between-run precision as well as accuracy results for all investigated QC levels are presented in Table 3. Guideline-compliant results were obtained for all kinin peptides^32^. In line with the maximally allowed deviation of 20% for accuracy and precision at the LLOQ, the LLOQ was set to 4.4 pg/mL for kallidin (S/N: 94), to 6.7 pg/mL for bradykinin (S/N: 96), to 10.6 pg/mL for des-Arg(10)-kallidin (S/N: 199), to 7.3 pg/mL for (des-Arg(9)-bradykinin (S/N: 403), to 6.5 pg/mL for bradykinin 1-7 (S/N: 155), to 8.1 pg/mL for bradykinin 2-9 (S/N: 65) and to 22.8 pg/mL for bradykinin 1-5 (S/N: 39). Representative chromatograms for the blank, the respective LLOQ, and the high QC are displayed in Fig. 4. The LoD was calculated as 3.5 pg/mL for kallidin, 2.5 pg/mL for bradykinin, 2.5 pg/mL for des-Arg(10)-kallidin, 3.0 pg/mL for des-Arg(9)-bradykinin, 4.4 pg/mL for bradykinin 1-7, 5.6 pg/mL for bradykinin 2-9 and 13.6 pg/mL for bradykinin 1-5.

**Table 3:** Accuracy and precision results for all analytes.

Analyte	Nominal concentration [pg/mL]		Accuracy				Precision	
			Day 1 RE [%]	Day 2 RE [%]	Day 3 RE [%]	Between-run RE [%]	Within-run CV [%]	Between-run CV [%]
Kallidin	QC high	3379.4	3.56	− 0.78	2.67	1.81	2.85	3.40
	QC mid	281.6	4.88	10.00	3.96	6.28	4.56	5.10
	QC low 4	70.4	− 2.52	5.28	− 7.64	− 1.63	4.56	7.77
	QC low 3	35.2	− 8.37	5.33	− 5.18	− 2.74	6.87	9.60
	QC low 2	17.8	− 3.79	5.86	− 3.42	− 0.45	7.55	8.70
	QC low 1	8.9	− 0.33	5.34	− 9.53	− 1.51	9.98	11.74
	LLOQ	4.4	8.29	16.40	− 2.99	7.23	13.50	15.11
Bradykinin	QC high	5145.9	1.09	3.57	2.89	2.52	4.16	4.16
	QC mid	428.8	4.30	5.01	4.28	4.53	5.05	5.05
	QC low 4	107.2	2.50	3.31	− 2.75	1.02	4.13	4.92
	QC low 3	53.6	− 1.30	4.28	− 6.91	− 1.31	3.82	6.62
	QC low 2	27.2	− 1.23	4.31	− 10.03	− 2.32	6.28	9.29
	QC low 1	13.6	− 0.39	2.29	− 8.99	− 2.36	9.10	10.13
	LLOQ	6.7	11.70	2.40	− 4.48	3.21	9.10	11.33
Des-Arg(10)-kallidin	QC high	4011.7	5.29	2.22	3.88	3.79	3.76	3.76
	QC mid	334.3	9.72	3.24	6.15	6.37	3.81	4.58
	QC low 4	83.6	2.25	− 1.47	− 0.37	0.14	6.45	6.45
	QC low 3	41.8	1.25	− 1.48	− 4.57	− 1.60	4.46	4.97
	QC low 2	21.2	5.61	2.30	− 6.55	0.45	7.23	9.00
	LLOQ	10.6	7.09	10.40	− 7.32	3.39	10.11	12.84
Des-Arg(9)-bradykinin	QC high	5564.3	− 0.79	2.54	0.27	0.67	4.14	4.14
	QC mid	463.7	7.82	8.80	12.01	9.54	5.07	5.07
	QC low 4	116.0	0.96	7.96	7.22	5.38	3.99	5.11
	QC low 3	57.9	− 0.45	3.95	1.55	1.68	3.02	3.46
	QC low 2	29.4	0.69	3.21	− 6.48	− 0.86	8.58	9.20
	QC low 1	14.7	− 0.02	0.04	− 13.40	− 4.46	9.83	11.96
	LLOQ	7.3	11.29	9.25	− 7.27	4.42	7.59	11.88
Bradykinin 2-9	QC high	6191.6	2.37	1.51	4.25	2.71	4.77	4.77
	QC mid	516.0	7.05	5.54	10.58	7.72	4.80	4.92
	QC low 4	129.0	6.91	5.60	0.61	4.38	5.46	5.83
	QC low 3	64.5	7.02	3.24	− 3.07	2.40	8.25	8.90
	QC low 2	32.7	10.37	6.87	− 12.36	1.63	9.64	14.81
	QC low 1	16.3	11.11	1.17	− 3.97	2.77	11.28	12.55
	LLOQ	8.1	15.14	15.50	− 8.02	7.54	12.97	17.08
Bradykinin 1-7	QC high	5001.8	3.06	14.11	0.18	5.78	7.31	9.54
	QC mid	416.8	10.80	10.66	8.45	9.97	8.01	8.01
	QC low 4	104.2	5.22	8.32	14.89	9.48	4.16	5.85
	QC low 3	52.1	7.78	6.04	2.81	5.54	7.47	7.47
	QC low 2	26.4	6.46	6.98	− 7.74	1.90	8.77	11.35
	QC low 1	13.2	8.43	1.98	0.12	3.51	6.53	7.20
	LLOQ	6.5	16.57	3.21	0.27	6.68	12.92	14.14
Bradykinin 1-5	QC high	4317.8	0.22	7.46	− 0.19	2.50	9.71	9.71
	QC mid	359.8	10.15	14.57	− 0.95	7.92	9.01	10.95
	QC low 4	90.0	2.10	12.37	7.64	7.37	10.37	10.44
	QC low 3	45.0	9.55	13.09	11.80	11.48	12.38	12.38
	LLOQ	22.8	6.65	12.08	13.82	10.85	14.92	14.92

Using one-way ANOVA, within-run precision was calculated as the repeatability and between-run precision as the day-different intermediate precision.

CV: coefficient of variation, LLOQ: lower limit of quantification, QC: quality control, RE: relative error.

The carry-over following the injection of an ULOQ sample was below 20% of the signal of the LLOQ for all analytes (kallidin: 19.0%, bradykinin: 17.0%, des-Arg(10)-kallidin: 16.8%, des-Arg(9)-kallidin: 19.1%, bradykinin 1-7: 11.9%, bradykinin 2-9: 18.9%, bradykinin 1-5: 4.4%). No carry-over was observed for the internal standard.

**Figure 5:**
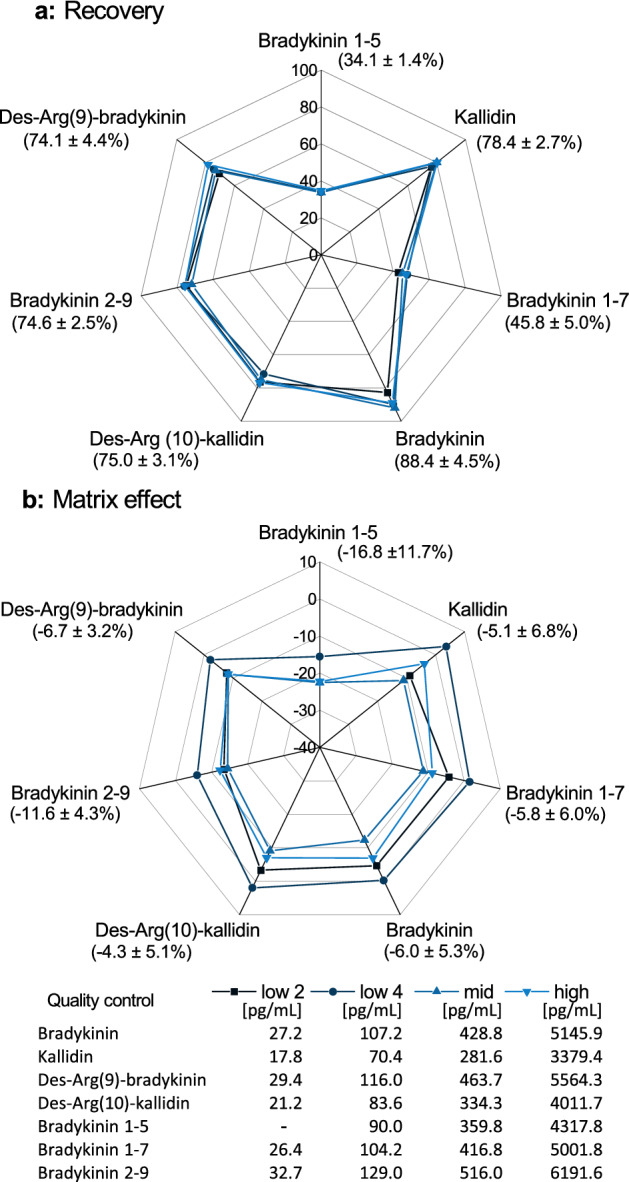
Recovery (**a**) and absolute matrix effect (**b**) of all analytes at the four investigated levels using saline matrix. Mean values and their coefficients of variation are presented in round brackets. Nominal concentrations of the quality control levels are depicted in the amended table.

At the distinct QC levels, recoveries did not vary substantially, as indicated by the low CVs of ≤ 5% between the different levels. The more hydrophilic analytes bradykinin 1-5 (mean 34.1%) and bradykinin 1-7 (mean 45.8%) presented lower recoveries compared to the peptides with more lipophilic or additional amine functional groups (mean 74.1% to 88.4%) (Fig. 5). Mean ion suppression of the four levels ranged from − 16.8% (bradykinin 1-5) to − 4.3% (des-Arg(10)-kallidin) (Fig. 5).

All kinin peptides were stable during autosampler storage for 18 h at 18 °C and throughout the short-term stability test for 24 h at 4 °C (supplementary table S2). With the exception of bradykinin 1-5, all other peptides were further stable for three freeze–thaw cycles and on the benchtop for 3 h. Bradykinin 1-5 was only stable for one hour on the benchtop, whereas after 3 h a mean decrease of − 31.0% was observed. Additionally, bradykinin 1-5 showed a tendency to degrade during freeze–thaw cycles, with increased degradation after three cycles compared to one (mean decrease: − 24.3% (1 h) vs. − 31.3% (3 h)). However, bradykinin 1-5, as well as the other kinin peptides, were stable for 15 freeze–thaw cycles measured over the course of 1 month (CV: 13.8%) in the analyte working solution. Since enzyme-free matrix was applied, the degradation was not related to insufficient inhibition of enzyme activity. Therefore, degradation might be caused by ionic interactions known to potentially affect instability. Thus, patient samples should be exposed to as few as feasible freeze–thaw cycles and be prepared freshly whenever possible.

### Applicability

**Figure 6:**
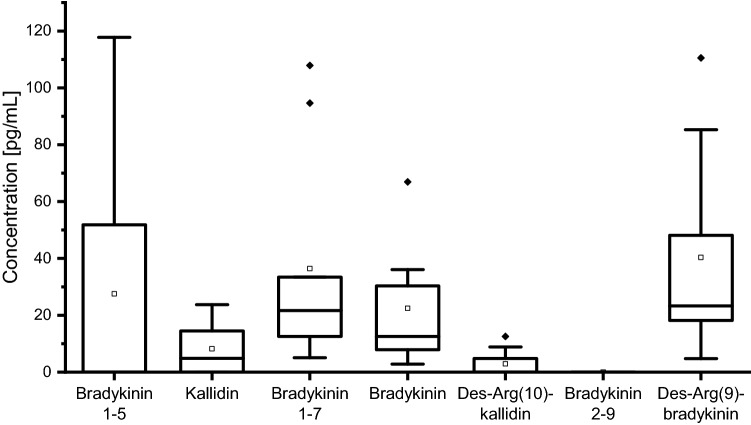
Box plots of the kinin peptide concentrations of healthy volunteers (n = 9) in nasal lavage fluid. Data were normalized to the recovered volume of nasal instillation with 10 mL saline. Filled black square: interquartile range, T: 1.5 interquartile range, –: median, open square: mean, filled black rhombus: outlier.

Endogenous levels of the kinin peptides in nine healthy volunteers were comprehensively determined in NLF and confirmed the method applicability. The recovery of instilled lavage volumes ranged from 33 to 89% in the healthy volunteers. Normalized mean levels for all kinin peptides were in the low pg/mL range; namely, 8.2 pg/mL for kallidin, 2.9 pg/mL for des-Arg(10)-kallidin, 22.5 pg/mL for bradykinin, 40.3 pg/mL for des-Arg(9)-bradykinin, 36.4 pg/mL for bradykinin 1-7 and 27.5 pg/mL for bradykinin 1-5 were measured. Bradykinin 2-9 was below the LoD (< 5.6 pg/mL) in all volunteer samples. Box plots of the level data are presented in Fig. 6 and a representative chromatogram is shown in Fig. 4.

## Discussion

The presented novel LC–MS/MS assay enabled the comprehensive and accurate determination of bradykinin, kallidin, des-Arg(9)-bradykinin, des-Arg(10)-kallidin, bradykinin 2-9, bradykinin 1-7, and bradykinin 1-5 in NLF. Characterized by a high sensitivity (4.4–22.8 pg/mL depending on the kinin) despite the use of low volumes, the applicability of this method was successfully proven by determining low-abundance kinin peptides in NLF. Full validation according to regulatory bioanalytical guidelines was achieved^32^.

To the best of our knowledge, this study is the first report of the comprehensive determination of kinin peptides in respiratory saline lavage fluid. Previous determinations of kinins in NLF by immunometric approaches did not quantitatively differentiate between the kinins and were limited to bradykinin and kallidin^19,20^. Immunoassays are prone to cross-reactivity with structurally similar peptides, which impacts the accuracy and reliability of the results. Further, these methods do not allow the simultaneous investigation and differentiation of kinin peptides from one sample aliquot. Thus, disease-related alterations in the KKS cascade by inhibition or inducement of enzyme activities that affect the generation or degradation of kinin peptides cannot be comprehensively assessed. Furthermore, data obtained from available LC–MS/MS methods is limited and provides a narrow scope of information. The restricted determination of not more than two kinin peptides simultaneously and the generally inadequate sensitivity to detect endogenous peptides in the low pg/mL range by LC–MS/MS has not yet allowed for a comprehensive assessment of the KKS.

Therefore, in advance of this method validation, extensive and systematic investigation was conducted to improve the sensitivity by optimizing the mobile phase and reducing nonspecific peptide adsorption of bradykinin^30^. By means of the DoE approach, substantial signal intensity increases for bradykinin—by a factor of 7.7 for the mobile phase optimization and by a factor of 26.6 for the injection solvent optimization—were achieved^30^. Following this approach, the intensity of the other peptides could now be improved through DoE and formed the basis to facilitate the low detection limits of 6.7 pg/mL for bradykinin and the range of 4.4 to 22.8 pg/mL for the other six kinin peptides using saline matrix. As suitable assays in saline solution are lacking, the performance of the developed assay can only be discussed in relation to other human matrices. For bradykinin 1-5, Seip et al. 2015 obtained a similar LLOQ of 20.3 pg/mL (vs. 22.8 pg/mL in the here presented study), but applied larger sample volumes (1 mL blood)^35^. The measurement of des-Arg(9)-bradykinin by LC–MS/MS was marked by a quantification limit of 2 ng/mL^25^. The LC–MS/MS assay of Lindström et al., established a LLOQ of 106.2 pg/mL for bradykinin using 500 µL of plasma^23^, which was already a factor of 100 below previously published LC–MS/MS methods with detection limits of 10 ng/mL^24,25^. However, this sensitivity was not sufficient to determine endogenous levels of bradykinin throughout all their plasma samples^23^.

In the current study, for the first time in NLF, concentrations of endogenous levels of specific kinin peptides were detectable in saline matrix and allowed for their comprehensive determination. Proud et al. 1983, measured kinin peptides in eight controls by immunometric detection, seven had levels below the LLOQ (< 20 pg/mL) and one had a level of 100 pg/mL. As mentioned above, a quantitative breakdown of the total kinin concentration compared to the respective peptides could not be made^19^. Turner et al., determined kinin levels of 68 (43–183) pg/mL (combined bradykinin and kallidin without distinction) (median (80% central range), n = 8)^20^. This is in line with the measured levels of healthy volunteers in NLF, where distinguished mean levels of 22.5 pg/mL for bradykinin and 8.2 pg/mL for kallidin were obtained. Levels for bradykinin 1-7, des-Arg(9)-bradykinin, des-Arg(10)-kallidin and bradykinin 1-5 were also in the low pg/mL range, as expected. Whereas bradykinin, bradykinin 1-7 and des-Arg(9)-bradykinin were detectable in all samples, presence of the other kinins in NLF varied individually and bradykinin 2-9 was below the quantification limit in all samples. Because levels of the cleaved peptides are not published elsewhere, a reliable classification of the concentrations is only possible to a limited extent, and it is rather necessary to ascertain these endogenous levels in larger healthy and diseased cohorts in future studies. Levels in patients are expected to exceed those in healthy volunteers if the hypothesis of a dysregulated KKS in COVID-19 can be confirmed. Therefore, the broad calibration curve range of the developed assay, covering a span of a factor of 250–1000 depending on the analyte, is expected to be suitable because it allows measuring endogenous levels in healthy controls, as well as detecting possible elevations. Further, application of the assay can easily be extended to other diseases in which alterations within the KKS are to be expected and provides the advantage of non-invasive and easy-to-handle sampling. Thus, it allows to investigate e.g. lung cancer, respiratory allergic reactions, and bradykinin-mediated side effects of ACE inhibitors^36–38^.

The developed LC–MS/MS assay further outmatched previously published immunoassay methods separating kinin peptides (bradykinin 1-7, des-Arg(9)-bradykinin, and bradykinin) in the low-abundant endogenous range regarding sample preparation effort, as it makes the final results available within 2 h of sampling. Campbell et al. 1993 applied a combination of C18 SPE followed by liquid–liquid extraction and chromatographic separation prior to immunoradiometric detection^21^. Duncan et al. purified their samples through five rounds of SPE followed by chromatographic separation before the fractions were analyzed by immunoassay^22^. Lower limits of quantification (0.3–0.4 pg/mL) were reached using these approaches; however, also 1 mL of blood was applied, which subsequently shows a sensitivity nearly equal to the presented LC–MS assay (100 µL sample volume). Advantages of the LC–MS assay are that falsification due to cross-reactivity can be excluded, and the obtained values are attributed to single peptides. Further, the significantly reduced sample preparation effort achieved in combination with a fast analysis time (~ 2 h vs. ~ 1 day), provides the opportunity to reduce the time working with potentially infectious patient samples.

Isotonic saline was chosen as the surrogate matrix for preparation of calibration curve and quality control samples to obtain blank matrix and avoid any interference with endogenous kinin peptides in respiratory lavage fluids. This was presumed to be an adequate approach, as NLF is mostly made up of saline owing to instillation with saline during lavage, with a reported dilution of factor 60–120^26^. Further, mucus, debris, and cells are separated by centrifugation. An important issue in quantitative lavage analysis is that the volume infused during saline lavage is not always equal to the volume sampled^39^. Therefore, when comparing data sets of determined peptide levels, e.g. at different time points or in different patients, normalizing against an endogenous dilution marker, such as albumin, total protein abundance, or urea has been proposed^40^. However, results might be influenced by capillary leakage in many respiratory disorders and an additional blood sampling is required to calculate the dilution^40,41^. Currently, no standardization regarding normalization of lavage fluids is available as stated in a consensus statement of the International Society for Heart and Lung Transplantation in 2020^42^. To maintain the non-invasive manner of the assay, in this study the recovered volume was used for normalization, which is the most commonly applied normalization strategy^42^. Because saline is used as surrogate matrix, the assay is not limited to the investigation of respiratory saline lavage fluids in humans, but can also be used in animal models. Thereby, the innovative assay allows to investigate the pathophysiology of COVID-19 but might also support the identification of possible new therapeutic targets if the hypothesis of an altered KKS can be confirmed in COVID-19.

In conclusion, the novel LC–MS/MS assay facilitates the comprehensive determination of kallidin, bradykinin, des-Arg(10)-kallidin, des-Arg(9)-bradykinin, bradykinin 1-7, bradykinin 2-9 and bradykinin 1-5 for the first time in saline. The method is well-suited for research purposes considering its high sensitivity and broad calibration curve range in combination with low applied volumes. The successfully validated method will contribute to elucidate the pathophysiology of SARS-CoV-2 by facilitating the investigation of the postulated connection between a dysregulated KKS and other clinical syndromes (e.g. COVID-19).

## Supplementary Information

Supplementary Information.

## Data Availability

The (raw) datasets in the context of the here presented validation are made available on a public repository (https://doi.org/10.5281/zenodo.4431745).
