# Supplementary material for: Sensitive mass spectrometric determination of kinin-kallikrein system peptides in light of COVID-19
Source: Sci Rep. 2021 Feb 4;11:3061. doi: 10.1038/s41598-021-82191-7 (PMC7862273; doi:10.1038/s41598-021-82191-7)
Supplement: Supplementary file 1 — Supplementary Information. [file 41598_2021_82191_MOESM1_ESM.pdf]

Supplementary Information

Sensitive mass spectrometric determination of kinin-kallikrein system peptides in light of COVID-19

Tanja Gangnus[1], Bjoern B. Burckhardt[1,*]

[1] Institute of Clinical Pharmacy and Pharmacotherapy, Heinrich Heine University, Universitaetsstr. 1, 40225 Dusseldorf, Germany

*corresponding author: bjoern.burckhardt@hhu.de

Table of contents:

Figure S1: Example calibration curves for all investigated kinin peptides.

Table S2: Results of stability studies for the kinin peptides.

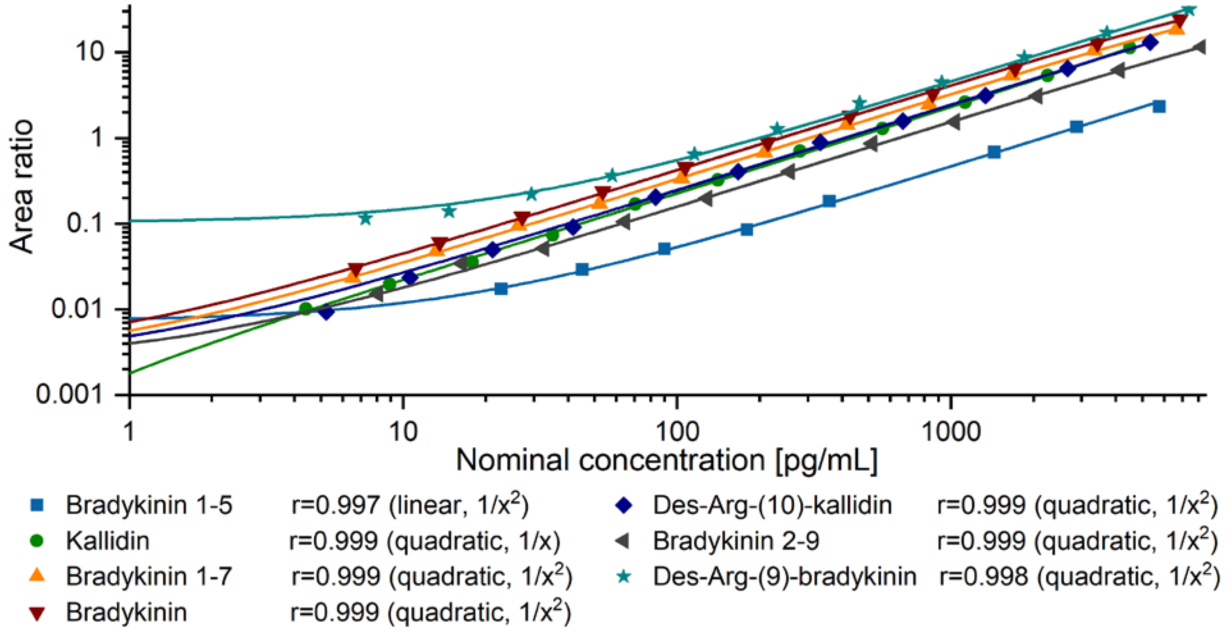

Figure S1: Example calibration curves for all investigated kinin peptides.

Table S2: Results of stability studies for the kinin peptides.

Stability:			Benchtop		Autosampler	Freeze-thaw		Short-term	
**Analyte**	**Nominal concentration [pg/mL]**		*3 hours*		*18 hours*	*3 cycles*		*24 hours*	
			RE [%]	*CV [%]*	*RE [%]*	*RE [%]*	*CV [%]*	*RE [%]*	*CV [%]*
**Kallidin**	QC low 2	17.8	-6.3	4.3	4.9	-0.9	10.9	3.0	6.4
	QC low 4	70.4	-7.6	2.2	7.1	-1.3	7.3	1.9	3.6
	QC mid	281.6	-8.1	2.6	5.0	-1.2	2.9	2.7	1.4
	QC high	3379.4	-9.0	3.0	13.1	0.3	2.7	7.8	2.0
**Bradykinin**	QC low 2	27.2	-6.4	8.9	13.8	5.9	5.7	8.7	7.8
	QC low 4	107.2	-10.7	5.5	9.4	2.6	6.2	13.4	1.9
	QC mid	428.8	-9.0	3.3	3.0	0.8	2.2	12.8	1.3
	QC high	5145.9	-7.3	3.8	7.7	3.2	2.8	14.7	2.0
**Des-Arg(10)-kallidin**	QC low 2	21.2	-6.2	6.5	9.1	4.4	10.5	14.0	10.2
	QC low 4	83.6	-9.4	3.2	8.3	0.5	7.0	11.6	4.4
	QC mid	334.3	-7.1	3.3	6.4	1.1	2.8	10.5	2.3
	QC high	4011.7	-4.9	3.5	7.7	-1.0	2.7	07.0	1.0
**Des-Arg(9)-bradykinin**	QC low 2	29.4	-14.8	11.4	-8.3	0.6	5.4	9.3	3.8
	QC low 4	116.0	-14.2	2.9	6.8	-1.6	5.4	10.4	2.6
	QC mid	463.7	-7.9	3.4	-0.2	-0.8	2.5	10.8	2.8
	QC high	5564.3	-13.3	2.5	4.6	-0.9	2.8	14.0	2.6
**Bradykinin 2-9**	QC low 2	32.7	-10.9	9.2	-6.3	9.0	3.9	13.7	7.7
	QC low 4	129.0	-13.5	7.9	10.5	1.5	4.4	12.2	4.7
	QC mid	516.0	-11.8	4.3	8.3	-3.8	1.6	4.1	2.4
	QC high	6191.6	-14.0	5.4	13.6	0.7	1.4	14.2	1.4
**Bradykinin 1-7**	QC low 2	26.4	-5.4	6.6	7.6	13.5	13.2	8.1	5.5
	QC low 4	104.2	-12.9	4.8	13.8	2.0	3.4	8.6	3.9
	QC mid	416.8	-9.1	4.3	9.1	-0.4	5.3	5.9	7.1
	QC high	5001.8	-10.3	3.5	8.3	-6.0	5.6	4.5	5.7
**Bradykinin 1-5**	QC low 4	90.0	-13.3^{+}	13.4 $^{+}$	14.2	-34.1*	24.9*	-0.3	7.7
	QC mid	359.8	-13.0 $^{+}$	17.7 $^{+}$	-1.8	-16.0*	9.0*	7.9	3.9
	QC high	4317.8	1.2^{+}	13.3 $^{+}$	9.8	-22.7*	9.8*	5.2	13.1

$^{+}$ 1h on benchtop * 1 freeze-thaw cycle, CV: coefficient of variation, QC: quality control, RE: relative error
